# Viscoelastic profiling of rare pediatric extracranial tumors using multifrequency MR elastography: a pilot study

**DOI:** 10.1038/s41598-026-55127-2

**Published:** 2026-05-28

**Authors:** Corona Metz, S. Veldhoen, H. E. Deubzer, F. Mollica, T. Meyer, K. Hauptmann, A. I. Heeren-Hagemann, A. Eggert, I. Sack, M. S. Anders

**Affiliations:** 1 Pediatric Radiology, Charité – Universitätsmedizin Berlin, corporate member of Freie Universität Berlin and Humboldt-Universität zu Berlin, Augustenburger Platz 1, 13353 Berlin, Germany; 2 Pediatric Hematology and Oncology, Charité – Universitätsmedizin Berlin, corporate member of Freie Universität Berlin and Humboldt-Universität zu Berlin, Berlin, Germany; 3 Radiology, Charité – Universitätsmedizin Berlin, corporate member of Freie Universität Berlin and Humboldt-Universität zu Berlin, Berlin, Germany; 4 Pathology, Charité – Universitätsmedizin Berlin, corporate member of Freie Universität Berlin and Humboldt-Universität zu Berlin, Berlin, Germany

**Keywords:** MR elastography, Viscoelastic properties, Oncology, Pediatric, Stiffness, Cancer, Medical research, Oncology

## Abstract

Magnetic resonance elastography (MRE) is a noninvasive technique for assessing viscoelastic properties of soft biological tissues in vivo, with potential relevance for tumor evaluation. This exploratory study aimed to assess the feasibility of multifrequency MRE in pediatric extracranial solid tumors and to investigate potential associations between viscoelastic parameters and different rare pediatric tumor entities. Ten pediatric patients (mean age, 5.7 ± 4.8 years; four female) with extracranial solid tumors underwent multifrequency MRE in this prospective study. Shear waves at 30–70 Hz were subsequently generated and measured with a phase-sensitive single-shot spin-echo planar imaging sequence. The obtained shear wave fields were processed by wavenumber (k-)based multi-frequency inversion to reconstruct tumor stiffness and fluidity. Viscoelastic properties within the tumors were quantified and correlated with the apparent diffusion coefficient (ADC). Differences in stiffness and fluidity were assessed across histopathologically confirmed tumor entities, stratified into malignancy-based groups. MRE was successfully performed in all patients within less than five minutes. Viscoelastic properties varied among tumor entities, with a tendency toward higher stiffness, fluidity, and spatial heterogeneity in tumors assigned to higher malignancy groups (all *p* < 0.05). Stiffness (*p* > 0.05) and fluidity (*p* < 0.05) showed inverse associations with tumor ADC values. Multifrequency MRE can be integrated into pediatric MRI examinations and provides quantitative information on tumor viscoelastic properties. Differences in stiffness and fluidity were observed across pediatric extracranial solid tumors with higher values in tumors assigned to higher risk groups. These preliminary findings suggest that MRE-derived parameters may provide complementary information for tumor characterization.

## Introduction

Magnetic resonance elastography (MRE) is a non-invasive imaging technique used to quantify viscoelastic properties of biological soft tissues in vivo^1^. Mechanical waves are generated, and motion-sensitive magnetic resonance imaging (MRI) sequences monitor the propagation of the resulting shear waves^2^. The measured shear wave characteristics are typically processed by wave inversion algorithms to generate viscoelastic parameter maps^3^. This technique provides high spatial resolution enabling detailed assessment of biomechanical properties without the need for invasive procedures, for example, in the evaluation of liver fibrosis or cirrhosis^4–6^.

Enzymes, such as lysyl oxidases, influence the formation of collagen fibers and their cross-linking. Increased cross-linking and thus stiffness of the tissue is observed in various fibrotic tissues and in desmoplastic reactions within tumors and is associated with aggressive cancer phenotypes^7,8^. Furthermore, it has been speculated that cancer cell motility influences the macroscopic tissue fluidity as measured by MRE in tumors^9^. Therefore, MRE could add diagnostic information to pediatric tumor differentiation, therapy response and outcome by determining the viscoelasticity of tumor tissue^10^.

Diffusion-weighted MRI (DWI) is routinely used in clinical routine to distinguish between benign and malignant lesions by assessing the diffusion behavior of water molecules within tissue^11^. By employing diffusion-sensitizing gradients, DWI captures microscopic water displacement over distances of approximately 1–20 μm. In oncologic imaging, restricted diffusion, typically due to increased cellular density and reduced extracellular space, is a hallmark of malignant tumors^12–14^. Given these characteristics, correlating DWI with MRE-derived parameters may provide complementary insights in tumor grading.

The diagnostic value of stiffness and fluidity as biomarkers in oncological imaging has been demonstrated in adults through pilot studies^15,16^. However, in the pediatric population, the potential of MRE remains largely unexplored. To date, only a few studies have investigated MRE in children, primarily focusing on the liver and the brain, with just one oncological study in children on intracerebral gliomas^17–20^. No data are available on MRE of extracranial solid tumors in children, leaving its diagnostic potential in this context undefined. This pilot study aimed to assess the feasibility of multifrequency MRE in pediatric extracranial tumors and to explore its diagnostic potential. Therefore, we present initial results of MRE in different pediatric tumor entities.

## Materials and methods

### Institutional review

The prospective study was approved by the institutional review board (Ethics Committee of Charité – Universitätsmedizin Berlin, approval number EA2/074/24). All methods were performed in accordance with the relevant institutional guidelines and regulations and with the Declaration of Helsinki. Written informed consent of the legal guardian was obtained prior to the examinations.

### Study sample and design

Inclusion criteria were newly or recently diagnosed extracranial pediatric solid tumors. Depending on the patient’s age and compliance, the examinations were performed under anesthesia (*n* = 5) or with the patient awake (*n* = 5). Exclusion criteria were refusal by the legal guardians (*n* = 0) and premature cancellation of the examination due to insufficient sedation (*n* = 1).

### Study population

Ten pediatric patients (mean age, 5.7 ± 4.8 years; median age, 4.7 years; age range, 4 month – 15 years; four female) with different tumor entities were included into this prospective single-center study between June and December 2024:

- One patient with abdominal alveolar rhabdomyosarcoma.
- One patient with Ewing’s sarcoma arising from the first rib.
- One patient with telangiectatic osteosarcoma of the left proximal tibial metaphysis.
- One patient with hepatoblastoma in the left liver lobe.
- One patient with nephroblastoma of the left kidney.
- Three patients with neuroblastomas:
  - One with high-risk neuroblastoma of the right adrenal gland.
  - One with cystic low-risk neonatal neuroblastoma (stage MS) in both adrenal glands.
  - One with low-risk neuroblastoma of the left adrenal gland.
- One patient with schwannoma on the proximal right forearm.
- One patient with lipoma on the right elbow.

All patients were examined using a 3-Tesla MRI scanner (Magnetom Skyra, Siemens Healthineers, Germany) using a 12-channel receiver coil.

Tumors were categorized into four risk groups (1–4) to enable a simplified comparison across entities. Risk stratification was based on a combination of biological factors, including tumor grading, MYCN amplification, proliferation rate, and gene expression profiles, as well as clinical features such as the presence of metastases, prognosis, expected therapy response, and recurrence rate^21–27^. Risk group 1 comprised benign tumors, while groups 2–4 represented malignant tumors with increasing clinical risk (low-, intermediate-, and high-risk, respectively). Given the heterogeneity of tumor types included in this study, this classification was applied as a harmonized framework for exploratory analysis. Tumors were manually segmented by a trained radiologist (C.M.) with six years of experience in radiology on gradient echo morphologic images. The resulting label maps were resampled to the MRE and DWI voxel grids using elastix^28^ with an identity transform and nearest-neighbor interpolation. No intensity-based image registration or spatial optimization was performed. Since all imaging modalities were acquired within the same examination session and patient positioning was maintained throughout the protocol, sufficient spatial correspondence between modalities was assumed for the purposes of this exploratory analysis.

### MRE

**Fig. 1:**
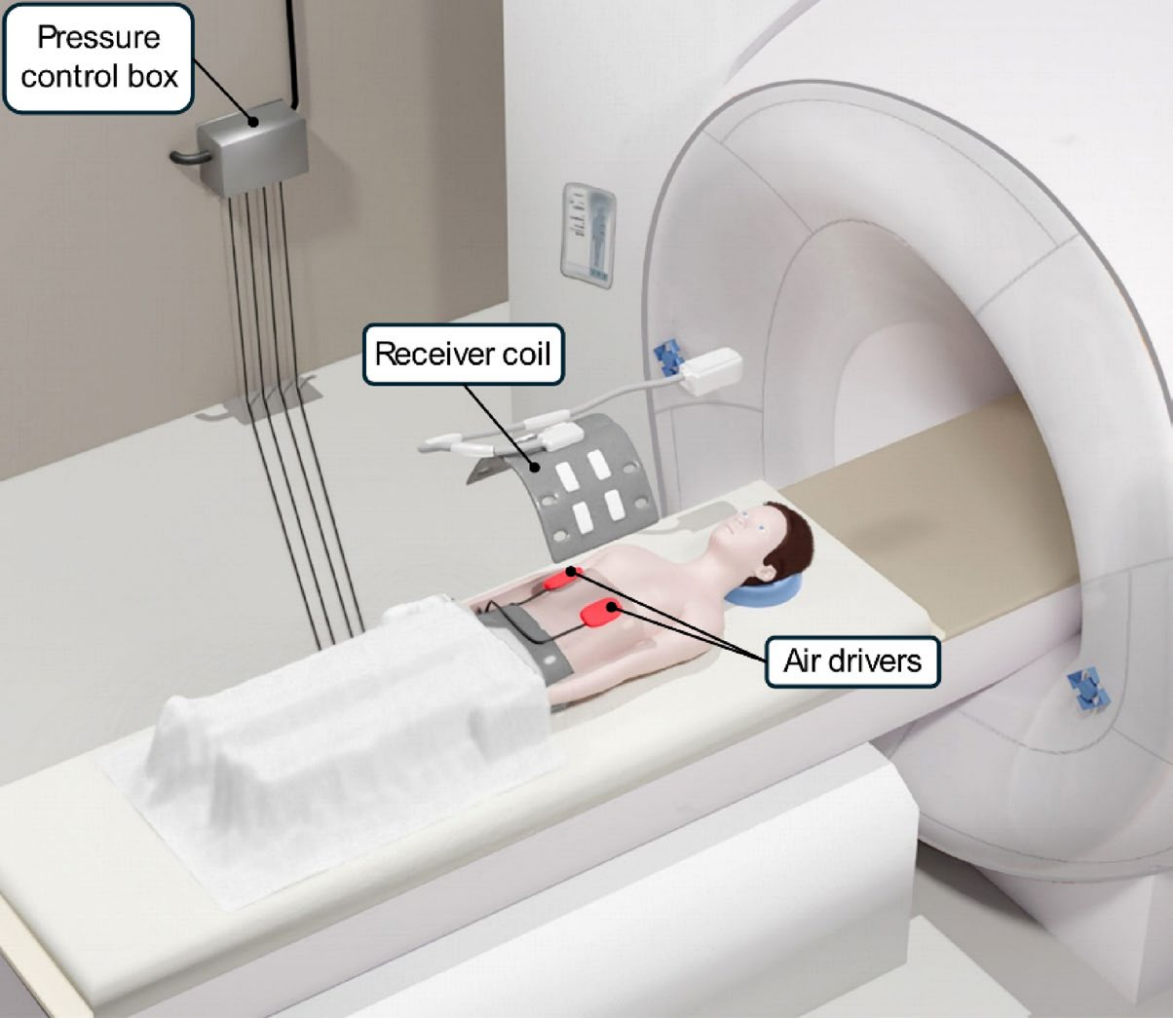
Pediatric MRE setup. The pressure control box controls the pressurized air flow used to periodically inflate and deflate four compressed air drivers at the desired frequency. The air drivers are attached near the region of interest using a Velcro belt (in the example shown, posterior and anterior of the abdomen). The resulting periodic tissue displacement alters the phase of the MR signal, which is captured by the radiofrequency receiver coil.

Vibrations were generated by four custom-made compressed air drivers attached to the patient using a Velcro belt positioned near the tumor localization. The compressed air flow was modulated to induce rhythmic in- and deflation of the drivers at the target frequency, resulting in time harmonic tissue displacements. Data acquisition was synchronized with vibrations by calibrating the internal clocks of the MRI system and the vibration generator. The pediatric MRE setup is shown in Fig. 1.

Vibrations were consecutively generated at 30, 40, 50, 60, and 70 Hz. Each corresponding wave period was sampled at eight equidistant temporal phases using a nullified first-order motion encoding gradient (MEG). The amplitude of the MEG was set to 40 mT/m with a duration of 15.20 ms, resulting in encoding efficiencies of 27.4, 17.1, 12.6, 10.3, and 9.4 rad/mm for the vibration frequencies of 30 to 70 Hz, respectively. MEGs were played out along the three Cartesian axes of the imaging coordinate system.

A total of 24 slices were acquired in a transverse orientation using a single-shot spin-echo echo-planar imaging sequence. Field of view (FOV) options of 180 × 140 or 280 × 224 mm² were selected based on patient size, with acquisition times of 4:20 or 4:31 min, respectively. Imaging parameters were defined as follows: Voxel size = 2.0 × 2.0 × 5.0 mm³; time of repetition (TR) = 2100 or 2150 ms; time of echo (TE) = 44 ms; generalized autocalibrating partially parallel acquisitions (GRAPPA) = 2; phase partial Fourier = 7/8.

The obtained MRE data were processed using the wavenumber (k-)based multi-frequency dual elasto visco inversion (k-MDEV), available as an online tool^3^, to reconstruct stiffness maps in terms of shear wave speed (SWS in m/s) and phase angle of the complex shear modulus (φ in rad), also termed tissue fluidity. Viscosity-related tissue fluidity reflects the viscous component of tissue mechanical behavior, which is not equivalent to tissue fluid content.

### Quality control for viscoelastic parameter estimation

Measurement success was assessed using the displacement signal-to-noise ratio (D-SNR), averaged across all applied excitation frequencies. Wave amplitudes were extracted from the magnitude of the complex-valued shear wave displacement fields, while noise levels were estimated using wavelet-based analysis of the displacement data^29^. The noise standard deviation was calculated as:

$$\:{\widehat{\sigma\:}}_{n}=\frac{\mathrm{m}\mathrm{e}\mathrm{d}\mathrm{i}\mathrm{a}\mathrm{n}(\mid\:{W}_{j}\mid\:)}{0.6745}$$

where $\:{W}_{j}$denotes the finest-scale wavelet detail coefficients. D-SNR was then defined as:

$$\:\mathrm{D}\mathrm{-}\mathrm{S}\mathrm{N}\mathrm{R}=20\cdot\:{\mathrm{l}\mathrm{o}\mathrm{g}}_{10}\left(\frac{{\sigma\:}_{\mathrm{w}\mathrm{a}\mathrm{v}\mathrm{e}}}{{\widehat{\sigma\:}}_{n}}\right)$$

where $\:{\sigma\:}_{\mathrm{w}\mathrm{a}\mathrm{v}\mathrm{e}}$represents the standard deviation of the displacement field amplitudes. Measurements with a mean D-SNR above 38.18 dB were considered acceptable for further analysis. The applied D-SNR threshold of 38.18 dB does not represent a universal quality scale with predefined categories. Rather, this threshold was empirically derived from displacement measurements obtained under no-excitation conditions, representing noise-dominated signal behavior in the absence of propagating shear waves^30^. Accordingly, values above this threshold indicate signal-dominated voxels with sufficient wave information for reliable viscoelastic parameter estimation. For statistical analysis, only regions with displacement amplitudes greater than 3.6 μm were included, as they indicate accurate estimation of the underlying viscoelastic properties^30^.

### DWI

DWI was performed using a gradient-echo echo-planar imaging sequence. Data were acquired at b-values of 50 and 800 s/mm² with 4 and 16 repetitions, respectively, using 12 diffusion-encoding directions. Depending on the size of the patient and the body parts examined, the FOV ranged from 168 × 280 to 310 × 382 mm² with 42–120 slices. Imaging parameters were defined as follows: Voxel size = 1.5 × 1.5 × 4.0 mm³; TR = 2000 ms; TE = 55–108 ms; Simultaneous Multi Slice Factor = 2.

### Statistics

Statistical analyses were performed using Matlab (version R2024a, MathWorks Inc, United States). Spearman’s rank correlation was used to assess associations between viscoelastic parameters, ADC values, and tumor risk groups. Given the small sample, all statistical analyses were considered exploratory and hypothesis-generating. Correlation analyses were performed to assess potential associations between variables. P-values < 0.05 were considered statistically significant; however, emphasis was placed on effect sizes and observed trends rather than formal statistical significance. No correction for multiple comparisons was applied due to the exploratory nature of the study.

## Results

Multifrequency MRE was performed successfully and without complications in all patients.

Table 1 shows the study population, and tumor entities with associated stiffness, fluidity, ADC and measurement quality metrics. Tumors were located in various anatomical regions, including the abdomen, thorax, and extremities. All measurements yielded mean D-SNR values within the tumor regions that exceeded the threshold of 38.18 dB and were therefore classified as successful acquisitions. In all cases except for the Ewing´s sarcoma and the rhabdomyosarcoma, at least 99% of voxels within the demarcated tumor regions exceeded the displacement amplitude threshold of 3.6 μm. The rhabdomyosarcoma exhibited over 96% of voxels above this threshold, while the Ewing sarcoma showed a markedly lower proportion, with only 54% of voxels meeting the criterion.

**Table 1:** Study population, tumor characteristics, and obtained imaging parameters.

Tumor type	Location	Risk group	Age in years / months	Sex	SWS in m/s	φ in rad	D-SNR	DA in µm	Reliable Voxel in %	ADC in ×10–3 mm²/s	Comment
Rhabdomyosarcoma	Intraabdominal	4	9 / 10	male	3.20 ± 1.16	0.84 ± 0.32	53.9 ± 0.4	6.6 ± 3.8	95	0.90 ± 0.45	Metastatic disease, treatment-naive
Ewing´s sarcoma	First left rib	4	6 / 8	female	2.30 + 0.62	1.12 ± 0.40	44.3 ± 0.9	4.1 ± 2.4	54	1.02 ± 0.53	Treatment-naive
High-risk neuroblastoma	Right adrenal gland	4	1 / 6	male	2.50 ± 1.21	1.15 ± 0.42	43.4 ± 1.8	5.9 ± 2.9	100	1.12 ± 0.43	Treatment-naive
Telangiectatic osteosarcoma	Left proximal tibial metaphysis	4	15 / 11	female	1.75 ± 0.71	0.74 ± 0.32	38.95 ± 4.9	8.5 ± 5.9	95	1.12 ± 0.63	Treatment-naive
Hepatoblastoma	Liver segments II-IV	3	1 / 9	male	2.36 ± 0.71	0.60 ± 0.25	46.3 ± 2.7	16.5 ± 8.1	100	1.50 ± 0.80	Neoadjuvant chemotherapy, treatment-naive
Nephroblastoma	Left kidney	3	2 / 11	female	2.15 ± 1.04	0.68 ± 0.28	45.2 ± 2.0	9.5 ± 6.2	99	1.38 ± 0.71	Metastatic disease, treatment-naive
Low-risk neuroblastoma	Left adrenal glands	2	1 / 6	female	1.92 ± 0.51	0.52 ± 0.24	54.8 ± 2.6	11.5 ± 7.4	100	1.18 ± 0.45	Treatment-naive
Low-risk neonatal neuroblastoma	Both adrenal gland	2	0 / 4	male	1.87 ± 0.59	0.68 ± 0.29	41.5 ± 1.4	11.0 ± 5.9	100	1.16 ± 0.60	Metastatic disease, Stadium MS, treatment-naive
Schwannoma	Right forearm	1	8 / 0	male	1.58 ± 0.31	0.54 ± 0.14	38.4 ± 3.8	21.1 ± 10.5	100	1.38 ± 0.34	Treatment-naive
Lipoma	Right elbow	1	8 / 1	male	1.33 ± 0.27	0.66 ± 0.25	0.66 ± 0.25	10.2 ± 5.5	10.2 ± 5.599	-	Treatment-naive

ADC: apparent diffusion coefficient; SWS: shear wave speed; φ: fluidity; D-SNR: displacement signal-to-noise ratio; DA: displacement amplitude. Imaging metrics are presented as mean ± standard deviation. No ADC value is provided for the lipoma, as diffusion-weighted imaging is unreliable in fatty lesions due to the low free water content and potential confounding effects from inadequate fat suppression. Risk groups were defined as follows: 1 = benign, 2 = low-risk malignant, 3 = intermediate-risk malignant, 4 = high-risk malignant tumors.

**Fig. 2:**
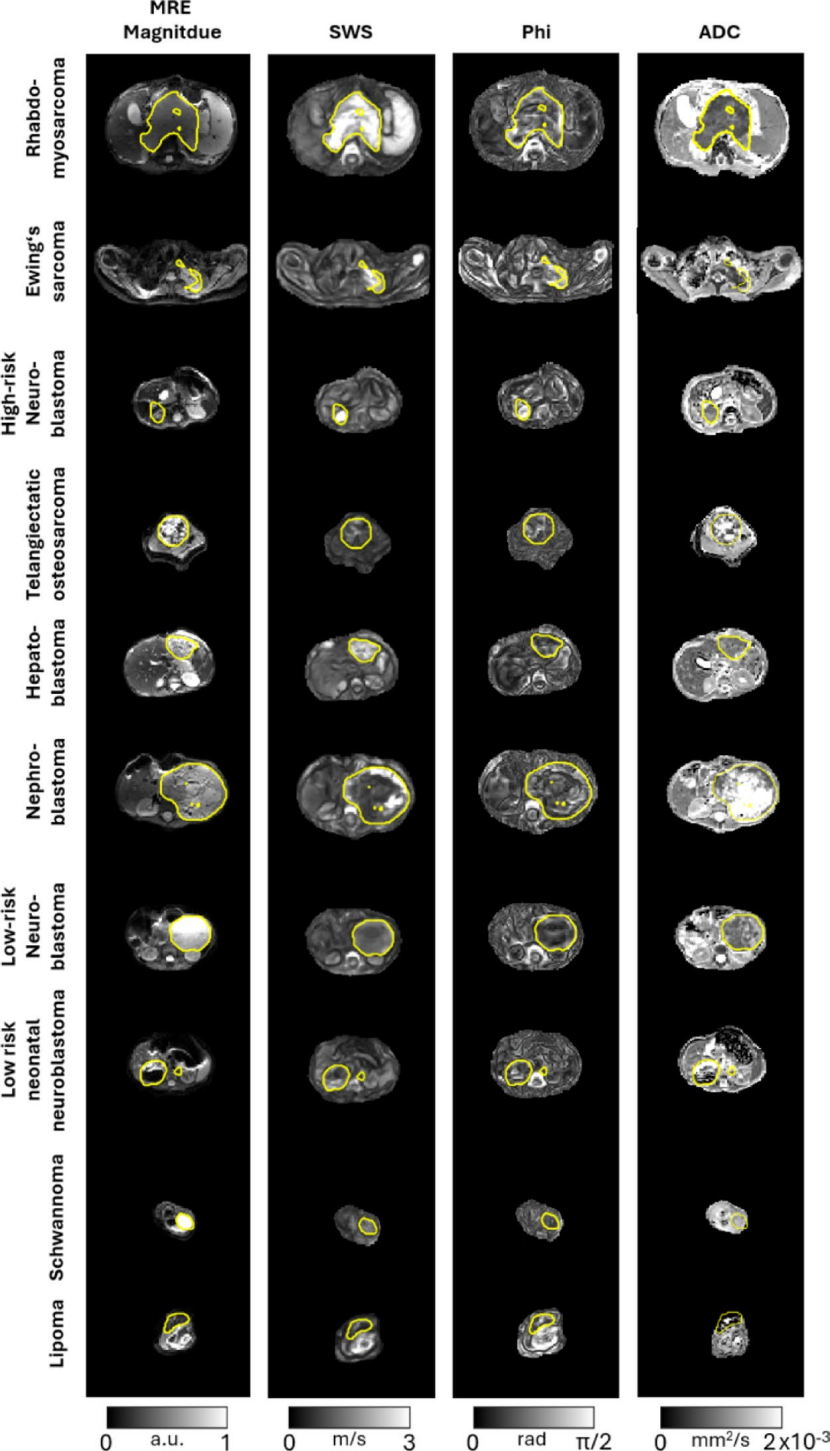
Representative slices of MRE magnitude, shear wave speed, viscosity-related fluidity, and ADC maps of each tumor. Tumor margins as delineated by a trained radiologist are shown, with regions exceeding the displacement threshold of 3.6 µm demacrated by yellow lines.

Fig. 2 shows representative slices of MRE magnitude, SWS, fluidity, and ADC for each tumor entity investigated. Yellow contours denote the tumor boundaries where sufficient shear wave displacement was present. The displacement fields show adequate wave propagation coverage within these regions, supporting the reliability of subsequent mechanical parameter estimation. Across tumor entities, a general trend is observed: more biological and clinical aggressive entities, such as rhabdomyosarcoma and high-risk neuroblastoma, exhibit higher SWS and lower ADC values, consistent with increased tissue stiffness and restricted diffusivity. In contrast, benign or low-malignancy tumors such as lipoma and low-risk neonatal neuroblastoma show lower SWS and higher ADC values.

**Fig. 3:**
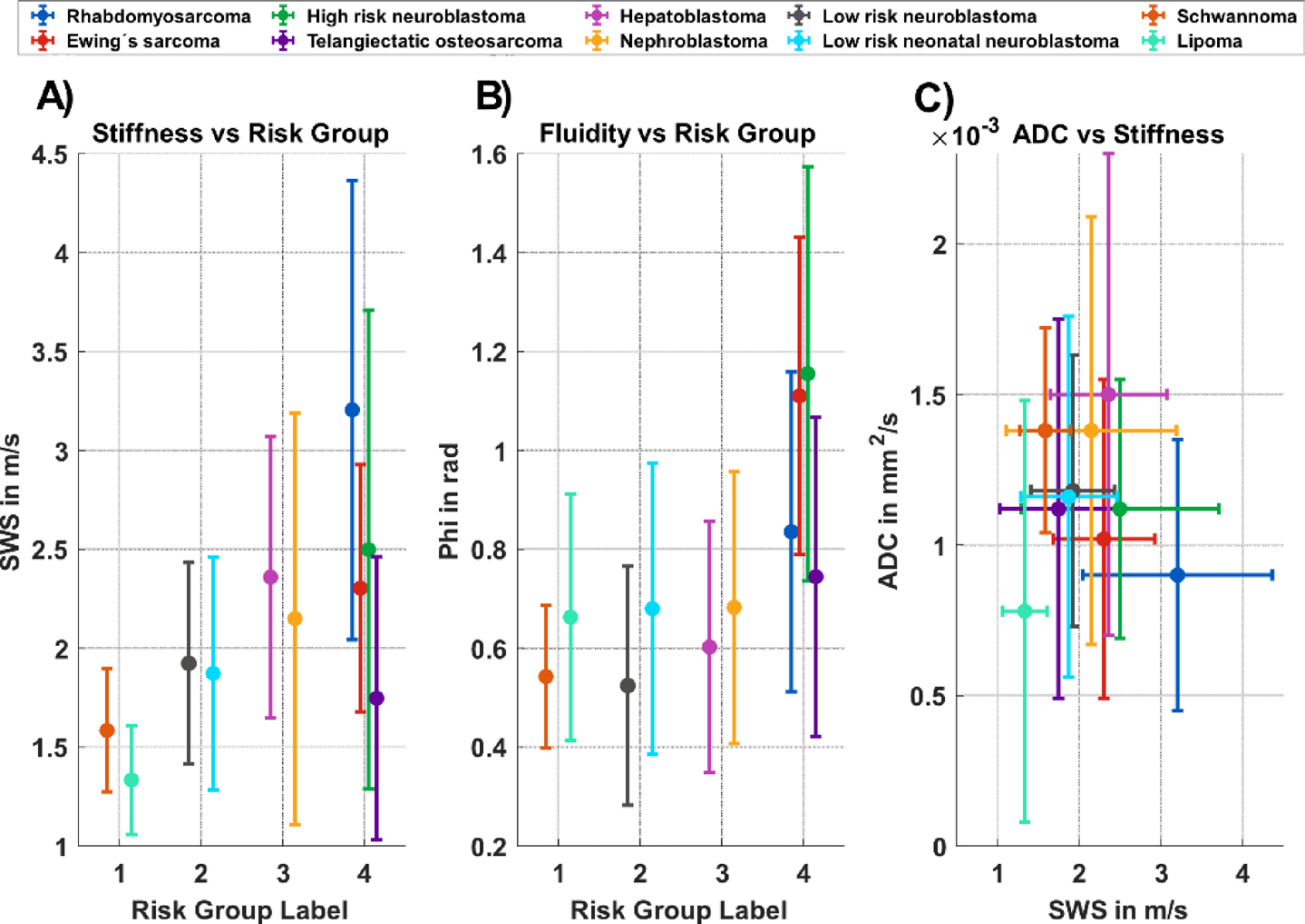
Viscoelastic and diffusion properties of pediatric tumors. Panels (A) and (B) show the distribution of stiffness and fluidity, respectively, across clinical and biological risk groups. Each data point represents an individual tumor case, with colors indicating different tumor entities. Panel (C) illustrates the relationship between stiffness and apparent diffusion coefficient (ADC) across the investigated tumors. Horizontal offsets in panels (A) and (B) were applied for visual clarity. Error bars represent the spatial standard deviation within the tumor volume and reflect intratumoral heterogeneity.

Fig. 3 presents SWS, fluidity, and ADC with their standard deviations, alongside tumor risk groups. Fig. 3a and b display SWS and fluidity across risk groups, respectively, while Fig. 3c shows ADC as a function of SWS. Each data point represents an individual tumor case. A tendency toward higher SWS, fluidity, and corresponding intratumoral variability can be observed in tumors assigned to higher risk groups.

Stiffness and fluidity were positively correlated with tumor risk group (ρ = 0.72, *p* = 0.014, *n* = 10) and (ρ = 0.84, *p* = 0.003, *n* = 10), respectively. When the telangiectatic osteosarcoma was excluded (significant differences in the viscoelastic properties of the tumor due to the large proportion of partially hemorrhaged cysts), stiffness and fluidity remained positively correlated with tumor risk group, whereas the correlation was higher for stiffness (ρ = 0.93, *p* = 0.001, *n* = 9) and lower for fluidity (ρ = 0.80, *p* = 0.014, *n* = 9).

Intratumoral viscoelastic heterogeneity also correlated significantly positively with tumor risk group, with stiffness heterogeneity (ρ = 0.85, *p* = 0.002, *n* = 10) and fluidity heterogeneity (ρ = 0.88, *p* = 0.001, *n* = 10). When the telangiectatic osteosarcoma was excluded, the correlation increased to (ρ = 0.88, *p* = 0.003, *n* = 9) for stiffness heterogeneity and was (ρ = 0.87, *p* = 0.005, *n* = 9) for fluidity heterogeneity. These results further support the association between viscoelastic properties and tumor aggressiveness.

An inverse association was observed between SWS and ADC (ρ = − 0.64, *p* = 0.097, *n* = 8) after exclusion of the lipoma and hepatoblastoma cases, as diffusion-weighted imaging is unreliable in fatty lesions and the treated state of the hepatoblastoma requires separate consideration (see Discussion). Fluidity showed a moderate inverse association with ADC (ρ = − 0.74, *p* = 0.046, *n* = 8). These findings should be interpreted with caution given the exploratory nature of the analysis and the small, heterogeneous cohort.

## Discussion

To the best of our knowledge, published data on MRE of pediatric extracranial solid tumors are extremely limited, with no prior dedicated cohort studies identified. This study therefore represents an initial exploratory investigation of viscoelastic tissue characteristics in pediatric extracranial solid tumors using MRE and provides preliminary observations on their mechanical properties across rare pediatric tumor entities.

All pediatric MRE scans surpassed the necessary displacement signal-to-noise ratio threshold of 38.18 dB within the tumor regions to ensure successful measurements. Most tumors showed near-complete voxel coverage above the displacement amplitude criterion of 3.6 μm, delineating robust shear wave propagation areas. The notably lower proportion of reliable voxels in the Ewing´s sarcoma is likely due to its deep anatomical location, where shear wave penetration is attenuated, leading to areas with reduced displacement amplitudes. This agrees with a known limitation of externally induced wave propagation in deeper or acoustically shielded regions^31^, though it did not affect overall diagnostic feasibility. The MRE quality metrics verified successful integration of the technique into the diagnostic workflow without complications for all pediatric patients, with acquisition times consistently under five minutes and no reported complications. Our findings are in good agreement with those of a large study by Joshi et al.^32^, in which 96% of MRE examinations in children and young adults were successfully completed.

The preliminary findings of our study suggest potential associations between viscoelastic properties and tumor risk group in pediatric extracranial tumors, as higher stiffness and fluidity values tended to be observed in tumors assigned to higher risk groups.

The rhabdomyosarcoma exhibited the highest stiffness and tissue fluidity, while the two benign tumors in our cohort—a schwannoma and a lipoma—showed the lowest values. Ewing’s sarcoma being less stiff than the rhabdomyosarcoma might be attributable to its anatomical location in the first left rib, where surrounding compliant thoracic tissues (muscle, lung, pleura) may mechanically buffer the tumor and reduce measurable stiffness. Additionally, Ewing’s sarcoma is known to be a highly vascularized tumor^33,34^, which may also contribute to lower stiffness and increased fluidity. SWS values of the hepatoblastoma being similar to the biologically very aggressive tumor types, may be artificially elevated due to chemotherapy-induced tumor regression and associated fibrotic remodeling. The telangiectatic osteosarcoma exhibited low stiffness and fluidity values compared to the other sarcomas and highly malignant tumors in our cohort. This may be explained by reduced extracellular matrix content, low cellular density, or limited stromal organization, all of which contribute to decreased mechanical integrity^35^. As a result, the measured viscoelastic properties may not accurately reflect the biological aggressiveness of this tumor entity but rather the physical properties of its cystic architecture. This is supported by stiffness correlating more closely with tumor risk group than fluidity when the telangiectatic osteosarcoma is excluded.

Cystic neuroblastoma and nephroblastoma, both of which contain substantial fluid-filled components, demonstrated stiffness and fluidity values higher than those of benign tumors but lower than those of other malignant lesions. High-risk neuroblastoma exhibited higher viscoelastic properties compared to the two low-risk neuroblastomas, which may be consistent with its more aggressive biological behavior. Low-risk neuroblastomas typically have an excellent prognosis and can, in some cases, such as stage MS, be managed with observation due to their potential for spontaneous regression^36^. In contrast, high-risk neuroblastomas are associated with poor outcomes and require multimodal therapy^37,38^. The two low-risk neuroblastomas differed only slightly, with the cystic lesion showing lower stiffness and higher fluidity, likely attributable to the physical properties of cystic fluids. These observations suggest that viscoelastic parameters may reflect differences in internal tumor composition in pediatric neuroblastoma.

Intratumoral viscoelastic heterogeneity also showed a positive association with tumor risk group. Increased heterogeneity may reflect underlying structural complexity, such as variations in cellularity, extracellular matrix composition, necrosis, or hemorrhagic components. In this context, the telangiectatic osteosarcoma in our cohort illustrates how pronounced internal heterogeneity, driven by cystic and hemorrhagic areas, can substantially influence measured mechanical properties. Although this resulted in lower overall stiffness, it highlights that spatial variability itself may carry biologically relevant information. Therefore, viscoelastic heterogeneity could represent a complementary parameter for identifying aggressive or structurally complex tumors, although this hypothesis requires validation in larger cohorts.

Similarly, the inverse relationship observed between SWS and ADC appears biologically plausible, as both parameters are influenced by tissue microstructure. Interestingly, fluidity also showed an inverse association with ADC. However, given the small and heterogeneous cohort, the biological interpretation of these findings remains uncertain and may be influenced by inter-entity differences and tumor composition. Potential confounding factors, such as tumor necrosis, cystic components, hemorrhage, and treatment-related changes, may influence both diffusion-weighted imaging and MRE-derived parameters. These factors can contribute to intralesional heterogeneity and may partially affect the observed mechanical and diffusion properties. Therefore, their potential impact should be considered when interpreting the presented results. The ADC map of the lipoma was excluded from analyses due to the unreliability of DWI in fat-rich tissues. This limitation arises from the low water content and distinct diffusion properties of fat, which result in weak diffusion signals and poor contrast. Additionally, fat suppression techniques—commonly employed to enhance DWI quality—may be insufficient in these tissues^39^.

In future studies, it may also be valuable to explore correlations between MRE-derived parameters and additional imaging biomarkers, such as those from dynamic contrast-enhanced (DCE) MRI, to gain further insights into tumor vascularity, perfusion dynamics, and their relationship to tissue biomechanics.

### Study limitations

The current findings represent preliminary data and individual observations. Further studies involving larger pediatric cohorts of certain tumor entities are needed to validate these results and expand on the utility of MRE in tumor characterization, treatment monitoring, and prognostication. Given the small sample size and heterogeneity of tumor entities, all statistical analyses should be interpreted as exploratory and hypothesis-generating rather than confirmatory.

Diffusion-weighted imaging was performed using two b-values (50 and 800 s/mm²), which differs from more extensive protocols commonly used in adult oncologic imaging that include higher b-values. While this approach reflects a clinically feasible compromise in pediatric imaging, it may reduce sensitivity to highly restricted diffusion.

Tumor segmentation was initially performed on morphological images and subsequently resampled to the MRE and DWI voxel grids using nearest-neighbor interpolation. Although all imaging modalities were acquired within the same examination session, minor spatial misalignment cannot be fully excluded, particularly due to differences in acquisition geometry and susceptibility-related distortions in DWI. These effects may be more pronounced in small lesions or at irregular tumor margins and could have influenced regional parameter assessment. In addition, segmentation was performed by an experienced radiologist, but reproducibility was not formally assessed in this study.

## Conclusion

Multifrequency MRE was successfully integrated into the routine diagnostic workflow of pediatric MRI and enabled quantitative assessment of viscoelastic tumor properties in rare pediatric tumor entities. Our preliminary results reveal that stiffness and fluidity vary across different pediatric tumor types and are associated with tumor risk group, with a tendency toward higher values in tumors assigned to higher risk groups. However, in tumors with large cystic or necrotic components, MRE measurements may be limited or biased and should therefore be interpreted with caution, at least until larger studies can more clearly define their diagnostic reliability in such contexts.

## Data Availability

The datasets used and/or analysed during the current study are available from the corresponding author on reasonable request.

## Abbreviations

ADC: Apparent diffusion coefficient
D-SNR: Displacement signal-to-noise ratio
DWI: Diffusion-weighted imaging
FOV: Field of view
GRAPPA: Generalized autocalibrating partially parallel acquisitions
MEG: Motion encoding gradient
MRE: Magnetic resonance elastography
SWS: Shear wave speed
TE: Time of echo
TR: Time of repetition
